# The Effect of MicroRNA-331-3p on Preadipocytes Proliferation and Differentiation and Fatty Acid Accumulation in Laiwu Pigs

**DOI:** 10.1155/2019/9287804

**Published:** 2019-12-04

**Authors:** Tao Chen, Jingxiang Cui, Lixia Ma, Yongqing Zeng, Wei Chen

**Affiliations:** ^1^College of Animal Science and Technology, Shandong Agricultural University, Tai'an 271018, Shandong, China; ^2^Shandong Provincial Key Laboratory of Animal Biotechnology and Disease Control and Prevention, Shandong Agricultural University, Tai'an 271018, Shandong, China; ^3^Weifang University of Science and Technology, Weifang 261000, Shandong, China

## Abstract

**Objective:**

The proliferation and differentiation of preadipocytes are regulated by microRNAs (miRNAs), hormones, and other factors. This study aimed to investigate the effects of miR-331-3p on the proliferation and differentiation of preadipocytes in addition to fatty acid metabolism.

**Methods:**

Preadipocytes were transfected with miR-331-3p mimics, miR-NC, or miR-331-3p inhibitor to explore its effect on cell proliferation and fatty acid accumulation. Furthermore, preadipocytes were transfected with pre-miR-331-3p, pcDNA3.1(+), or miR-331-3p inhibitor to explore its effect on differentiation.

**Results:**

It was observed that miR-331-3p could inhibit preadipocytes proliferation. Furthermore, miR-331-3p was highly expressed during cellular differentiation and appeared to promote the process. In addition, dual fluorescein analysis showed that dihydrolipoamide S-succinyltransferase (DLST) is a target gene of miR-331-3p, and overexpression of miR-331-3p could regulate the metabolism of fatty acids in the citrate pyruvate cycle by targeting DLST expression.

**Conclusion:**

In summary, these findings indicated that miR-331-3p exerts contrasting effects on the processes of fat deposition.

## 1. Introduction

The Laiwu pig is a Chinese breed with many characteristics that make it favorable for the commercial market. The meat has a high level of intramuscular fat, averaging approximately 10.32%. This is significantly higher than Yorkshire pigs (2% intramuscular fat) as well as many others Chinese breeds that have around 5% intramuscular fat on average. Although some studies have been carried out on the fat deposition of Laiwu pigs [1–3], the actual mechanism remains elusive.

The amount of fat deposited in an animal depends on body's balance of synthesis and catabolism rates. Recent studies have shown that animal fat deposition is the result of not only an increase in the number of fat cells but also an increase in the volume and accumulation of lipid droplets. Factors affecting the differentiation and growth of fat cells may also affect the formation of fat. Preadipocytes, which have the ability to proliferate and differentiate into adipocytes in vivo, have become an important model helping to complete the current understanding of adipose tissue formation and proliferation. It has been reported that the proliferation, differentiation, and fat deposition of preadipocytes are regulated by various factors [4].

In recent years, studies have shown that miRNAs are involved in the regulation of many biological processes, such as cell proliferation and differentiation, biological metabolism, and adipogenesis [5–7]. MicroRNAs mainly interact with the 3′-UTR of the target gene, which often leads to degradation of the target gene mRNA or inhibition of translation. In other words, they exert posttranscriptional regulation of target genes. Many miRNAs play an important role in the regulation of fat formation [8, 9]. For example, miR-143 promotes adipogenesis by acting on the target gene ERK5 [10, 11], miR-21 promotes adipogenesis by acting on the target genes TGFBR2 and STAT3 [12, 13], and miR-519d promotes fat deposition by acting on the target gene PPAR*α* [14]. It has also been demonstrated that both miR-27 and miR-130 inhibit adipogenesis by acting on the target gene PPAR*γ* [15–17]. Another miRNA that inhibits adipogenesis is miR-224, which exerts the effect by acting on the EGR2 gene [18]. However, there are still many mechanisms of miRNA action, and the role of miRNAs in adipocyte proliferation, differentiation, and lipid metabolism requires further research. With the development of high-throughput sequencing technology, an increasing number of miRNAs have been discovered to be involved in the fat metabolism pathway, thus promoting the study of miRNAs and their target genes involved in adipose tissue function. Xie et al. used the Illumina sequencing technology to analyze differential expression of miRNAs in the livers of Tongcheng and Yorkshire pigs [19]. They were able to identify 58 differentially expressed miRNAs. Furthermore, high-throughput sequencing was employed to analyze subcutaneous fat of 7- and 240-day-old Rongchang pigs. A total of 93 upregulated and 33 downregulated miRNAs were discovered at 240 days of age [20]. Similarly, Chen et al. also identified 9 differentially expressed miRNAs in Meishan pig back fat, indicating that miRNAs may regulate fat deposition in pigs [21].

Currently, numerous studies have reported that miR-331-3p plays an important role in the proliferation and differentiation of cancer cells, as well as the occurrence and development of cancer [22–24]. Furthermore, miR-331-3p is able to directly target the cell cycle-associated gene, E2F1. Upregulation of miR-331-3p has the ability to inhibit the growth and clonal formation of gastric cancer cells [25]. The expression of miR-331-3p in prostatic tissue is low. Transfection of miR-331-3p can inhibit the expression of ERBB-2 gene and inhibit the downstream PI3/Akt sex hormone receptor signaling pathway. It can inhibit the expression of prostate-specific antigen (PSA) by reducing the activity of androgen to stimulate the promoter of PSA. It is also found that the expression of miR-331-3p may be specific in the nervous system [26–28]. In addition, miR-331-3p has also been used as a marker for the detection of liver cancer in serum [29]. Sequencing of subcutaneous adipose tissue (by RNA-seq) of Laiwu and Yorkshire pigs showed that 17 upregulated and 22 downregulated miRNAs were identified in Laiwu pigs [30]. Chen et al. use both Laiwu and Yorkshire pigs as research subjects, and high-throughput sequencing analysis of the longissimus dorsi muscle of the 2 pig breeds was performed using the RNA-seq technology [31]. Consequently, 19 significantly differentially expressed miRNAs were detected and selected for further screening. Among them, relative to Yorkshire pigs, 7 differentially expressed miRNAs, including miR-331-3p, were upregulated, whereas 12 were downregulated in Laiwu pigs. In conclusion, miR-331-3p may influence the formation of adipose tissue as well as intramuscular fat deposition in pigs.

Through bioinformatics prediction, we found that DLST and solute carrier family 25 member 1 (SLC25A1) are the target genes of miR-331-3p, and these two genes are related to fat deposition. DLST encodes dihydrolipoyl succinyltransferase, which is a core component of KGDHC, which is the rate-limiting enzyme for the second oxidative decarboxylation of the Krebs cycle [32]. The SLC25A1 gene encodes a mitochondrial citrate transporter (CTP) that regulates citric acid transport bidirectionally between the mitochondria and cytoplasm [33]. Both DLST and SLC25A1 are present in the citrate pyruvate cycle pathway, which transports the acetyl-COA necessary for the synthesis of fatty acids from the mitochondria into the cytoplasm. In the present study, the interaction of DLST and SLC25A1 with miR-331-3p and their role in fat deposition were researched.

## 2. Materials and Methods

### 2.1. Experimental Animals and Sample Collection

Two breeds of pigs, Laiwu (weight = 100 kg, *n* = 6) and Yorkshire (weight = 100 kg, *n* = 6), served as the research subjects in this study. The pigs were raised in different groups at the Laiwu and Yorkshire farm (Laiwu city, Shandong, China) and were raised in the same environment. All groups were also given identical diets, which met their nutrient requirements as previously described (NRC, 1998). Subsequently, all pigs were ethically sacrificed and processed for market. Tissue samples including heart, liver, kidney, spleen, lung, muscle (longissimus dorsi), and fat (back fat) were collected and stored at −80°C. In addition, ear tissue samples from the Laiwu pigs were collected and stored at −20°C. All animal experiments were performed in accordance with the Institutional Animal Care as well as the National (GB 13078–2001 and GB/T 17237–1998) and the Agricultural Standards (NY 5148-2002-NY 5151-2002) of the People's Republic of China.

### 2.2. Cell Culture

PK15 cells (a porcine kidney epithelial cell line) were maintained in Dulbecco's modified Eagle's medium (DMEM; Gibco, USA) containing 10% fetal bovine serum (FBS; Gibco), penicillin (100 U/ml), and streptomycin (100 *μ*g/ml) at 37°C in a humidified 5% CO_2_ atmosphere. Cells used in experiments were in the logarithmic phase of growth and cell viability before treatment was >95%.

### 2.3. Isolation, Culture, and Differentiation of Laiwu Porcine Primary Preadipocytes

Laiwu pigs (<7 days of age) were obtained from the Laiwu and Yorkshire farm. Adipose tissue was isolated from porcine back and neck under sterile conditions. Adipose tissue was digested with 1 mg/mL collagenase 1 (Solarbio, Beijing, China) at 37°C for 60 to 90 min. Then, FBS-supplemented medium was added to terminate the digestion, and all the liquids were sifted through a 100 *μ*m cell strainer. The culture medium was removed by centrifugation (100 ×*g*, 10 min). Subsequently, Red Blood Cell Lysis Buffer (Solarbio, Beijing, China) was added and incubated at room temperature for 10 min and was centrifuged at 200 ×*g* for 10 min. Finally, the primary preadipocytes were resuspended in Dulbecco's modified Eagle's medium DMEM/F12 (Hyclone, Shanghai, China) containing 10% fetal bovine serum (FBS, Gibco, USA) and 1% penicillin-streptomycin (Pen-strep, Solarbio, Beijing, China).

Porcine preadipocytes were cultured in growth medium for 3 days to reach approximately 80% confluence (day 0). Then, the growth-arrested cells were cultured in differentiation medium (DMEM/F12, 10% FBS, 1% Pen-strep, 5 ug/mL insulin (Solarbio, Beijing, China), 1um/mL dexamethasone (DEX, Solarbio, Beijing, China), and 0.5 mM/mL 3-isobutyl-1-methylxanthine (IBMX, Solarbio, Beijing, China)) for 2 days (Day 2). The cells were then treated with maintenance medium (DMED/F12, 10% FBS, 1% Pen-strep, and 5 ug/ml insulin) for an additional 6 days. During this period, the maintenance medium was replaced every 2 days.

### 2.4. Bioinformatics Analysis

Target sequences for miR-331-3p were predicted using TargetScan (http://www.TargetScan.org), MiRBase (http://www.mirbase.org), and miRWalk (http://zmf.umm.uni-heidelberg.de/apps/zmf/mirwalk/).

### 2.5. Construction of 3′-UTR Luciferase Reporter Plasmids

Wild-type DLST and SLC25A1 3′-UTR fragments containing the binding site of miR-331-3p were amplified by PCR from total RNA extracted from the back fat of Laiwu pigs. Products of the PCR reactions were directionally subcloned into the pGL3 vector downstream of the firefly luciferase open-reading frame (Promega, USA). Mutation of the seed sequence (the miR-331-3p target site) was accomplished by overlap extension PCR using the general primer and mutagenic primer, which are listed in Table 1. First, the wild-type DLST and SLC25A1 3′-UTR fragments were used as templates, including miR-331-3p target site. The mutant DLST 3′-UTR fragment's upstream and downstream regions were amplified with primers F1, R3 and F3, R1 (the same as above), respectively. The mutant SLC25A1 3′-UTR fragments were amplified using primers F2, R4 and F4, R2. Next, the wild-type and mutant DLST and SLC25A1 3′-UTR fragments were both inserted into the pGL3 vector. Positive clones were identified through bacterial-liquid PCR and were confirmed by sequencing (Sangon Biotech, Shanghai, China). The wild-type and mutant DLST 3′-UTR vectors were named as pGL3-DLST-wt and pGL3-DLST-mut, respectively. The wild-type and mutant SLC25A1 3′-UTR vectors were named as pGL3-SLC25A1-wt and pGL3-SLC25A1-mut, respectively.

Preadipocytes were fixed for 1 h in 10% formalin, washed with PBS, and stained for 30 min by complete immersion in a working solution of Oil Red O. The cells were then washed twice in water, and data were collected by imaging the cells. In order to quantify fatty acids, excess water was evaporated by placing the stained cultures at a temperature of about 32°C for 15 min. Next, 1 ml of isopropyl alcohol was added to the stained culture dish, and the extracted dye was immediately removed. The absorbance of the resulting solution was monitored spectrophotometrically at 510 nm.

### 2.6. Dual-Luciferase Reporter Assay

PK-15 cells (Procell, Wuhan, China) cultured in 24-well plates were transfected using Lipofectamine™ 3000 Transfection Reagent (Thermo, USA) according to the manufacturer's instructions. The transfection mixture for each well contained 250 ng 3′-UTR luciferase reporter vector, 12.5 ng of pGL4.74 (Promega, USA) for normalization, and either 15 pmol miR-331-3p mimic expression plasmids, miR-331-3p mimic, or negative control mimic (GenePharmn, Shanghai, China). Each group of experiments was repeated at least 3 times. After 48 h of transfection, the luciferase activity was measured using the Dual-luciferase Reporter Gene Assay System Kit (Promega).

### 2.7. RNA Extraction and Gene Expression Assay Using Quantitative Real-Time PCR (qRT-PCR)

Total RNA was extracted from the cell and tissue samples using the MicroElute Total RNA Kit (Omega Bio-Tek, USA) according to the manufacturer's instructions. The quantity of the isolated RNA was determined by measuring the UV260/280 absorbance ratio using a biophotometer (Eppendorf, Germany). For miRNA reverse transcription, Mir-XTM miRNA First-Strand Synthesis Kit (Takara Biotechnology, Dalian, China) was used. Quantitative Real-Time PCR (qRT-PCR) was performed using the SYBR Premix Ex Taq Kit (Takara Biotechnology, Dalian, China) on a LightCycler® 96 (Roche). The miRNA-specific primers for the qRT-PCR included the forward primer GGTATGGGCCTATCCTAGAA and universal primer used for the reverse primer. The housekeeping gene U6 was used as an internal control.

For mRNA reverse transcription, PrimeScript™ RT Reagent Kit with gDNA Eraser (Takara Biotechnology, Dalian, China) was used according to the manufacturer's instructions. qRT-PCR was performed using the SYBR Premix Ex Taq Kit (Takara Biotechnology, Dalian, China) on a LightCycler® 96 (Roche). GAPDH (glyceraldehyde-3-phosphate dehydrogenase) was used as an internal control for the other genes. Primers used to amplify the mRNAs are listed in Table 2. The quantitative fluorescence results were calculated by 2^−ΔΔCt^ method.

### 2.8. Transfection of Porcine Preadipocytes

Preadipocytes were seeded in 96-, 24- or 6-well plates. Once cell densities reached 80% confluence, they were transfected with either the mimic, inhibitor, or negative control for miR-331-3p (Sangon, Shanghai, China), respectively. The miR-331-3p mimic and inhibitor were transfected with Lipofectamine 3000 (Invitrogen, Carlsbad, CA, USA) at concentrations of 3 pmol, 15 pmol, and 75 pmol/well for 4 h. After that, the growth medium was replaced.

### 2.9. Detection of Cell Proliferation Activity by Cell Counting Kit 8 (CCK-8 Assay)

Preadipocytes were inoculated into 96-well plates at a density of 2 × 10^3^. Five replicates were used for each group of cells, and 5 blank controls were used to remove background signals. After culture for 24, 48, 72, and 96 h, 10 *μ*L of CCK-8 reagent was added to each well and incubated for 2 h. The absorbance of each well was determined at 450 nm using a microplate reader (Labsystem). The cell proliferation curve was plotted, and the experiment was repeated in triplicate.

### 2.10. Flow Cytometry

Preadipocytes were seeded in 96-well plates and incubated for 24 h. By this time, the cells reached confluence and were transfected with either miR-331-3p mimic, miR-NC, or miR-331-3p inhibitor (GenePharma, Shanghai, China). Preadipocytes were harvested by digestion with 0.25% trypsin and washed 3 times with PBS to remove cell debris. The cells were then fixed in cold 70% ethanol overnight and treated with 1 mg/mL RNaseA and propidium iodide (PI) for 30 min at 37°C. The cells were then filtered through a 0.75 *μ*m membrane prior to analysis on a FACScan argon laser cytometer (BD, USA).

### 2.11. Western Blotting

Preadipocytes were washed with PBS, lysed using RIPA buffer (Beyotime, Shanghai, China) on ice, and centrifuged at 10,000 ×*g* at 4°C. The whole protein concentration for each sample was measured using the BCA Protein Assay Kit (Beyotime, Shanghai, China). All protein samples and SDS-PAGE Sample Loading Buffer (Beyotime, Shanghai, China) were mixed at a ratio of 4 to 1 and boiled for 5 min. Next, all protein samples were diluted to the same concentration. Protein bands were resolved by SDS-PAGE and transferred onto polyvinylidene difluoride (PVDF) membranes. The membranes were then blocked with 5% Blocking Buffer (Beyotime, Shanghai, China) for 1 h. Next, primary antibodies directed against SLC25A1, DLST, or actin (Cell Signaling Technology, CST, USA) were incubated on the membranes at 4°C overnight. The membranes were then treated with HRP-conjugated secondary antibodies (Cell Signaling Technology, CST, USA) at room temperature for 2 h. Signals were detected using the enhanced chemiluminescence system reagents (Amersham Pharmacia Biotech, USA). All experiments were repeated in triplicate.

### 2.12. Statistical Analysis

All data are presented as means ± standard deviation (SD). Statistical significance was assessed by Student's *t*-test. Differences were considered statistically significant at *p* < 0.05.

## 3. Results

### 3.1. Expression Analysis of miR-331-3p in Different Tissues of Laiwu and Yorkshire Pigs

**Figure 1:**
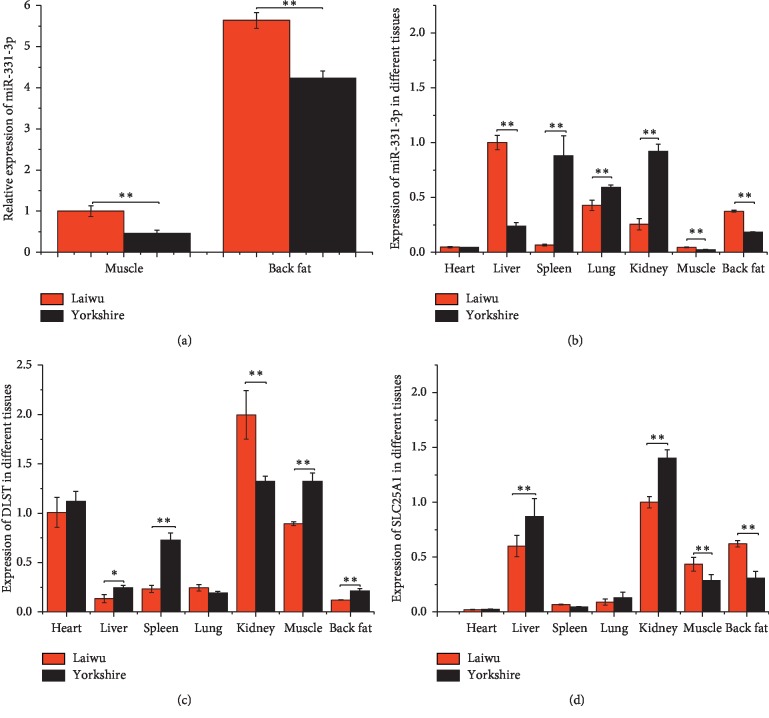
Expression levels of miR-331-3p, DLST, and SLC25A1 in Laiwu and Yorkshire. (a) qRT-PCR result of the expression level of miR-331-3p in muscle and back fat; results of the expression levels of miR-331-3p (b), DLST (c), and SLC25A1 (d) in different tissues, including heart, liver, spleen, lung, kidney, muscle, and back fat. U6 and GAPDH as housekeeping genes were used for the detection of miRNA and mRNA, respectively (*n* = 3) (^*∗*^*p* < 0.05; ^*∗∗*^*p* < 0.01).

To investigate the differences in miR-331-3p expression between Laiwu and Yorkshire pigs, RNA was extracted from various tissues, and the expression levels were tested by qRT-PCR (Figures 1(a) and 1(b)). Significant differences were observed between the 2 breeds. In the liver, muscle, and back fat of Laiwu pigs, miR-331-3p expression was more than that of Yorkshire pigs (*p* < 0.05). However, the opposite was observed in the spleen, lung, and kidney (*p* < 0.05).

Through a biological website analysis, 2 candidate target genes of miR-331-3p were predicted (DLST and SLC25A1). Expression levels of the 2 genes were assessed in various tissues between the 2 breeds of pigs by qRT-PCR. The expression trend of DLST gene is opposite to the expression trend of miR-331-3P, whether in Laiwu pigs' or in Yorkshire pigs' liver, kidney, muscle, and back fat (Figure 1(c)) (*p* < 0.05). However, except in liver tissue, the expression of SLC25A1 was significantly elevated in the muscle and back fat (Figure 1(d)) (*p* < 0.05).

### 3.2. miR-331-3p Suppressed Proliferation of Porcine Preadipocytes

**Figure 2:**
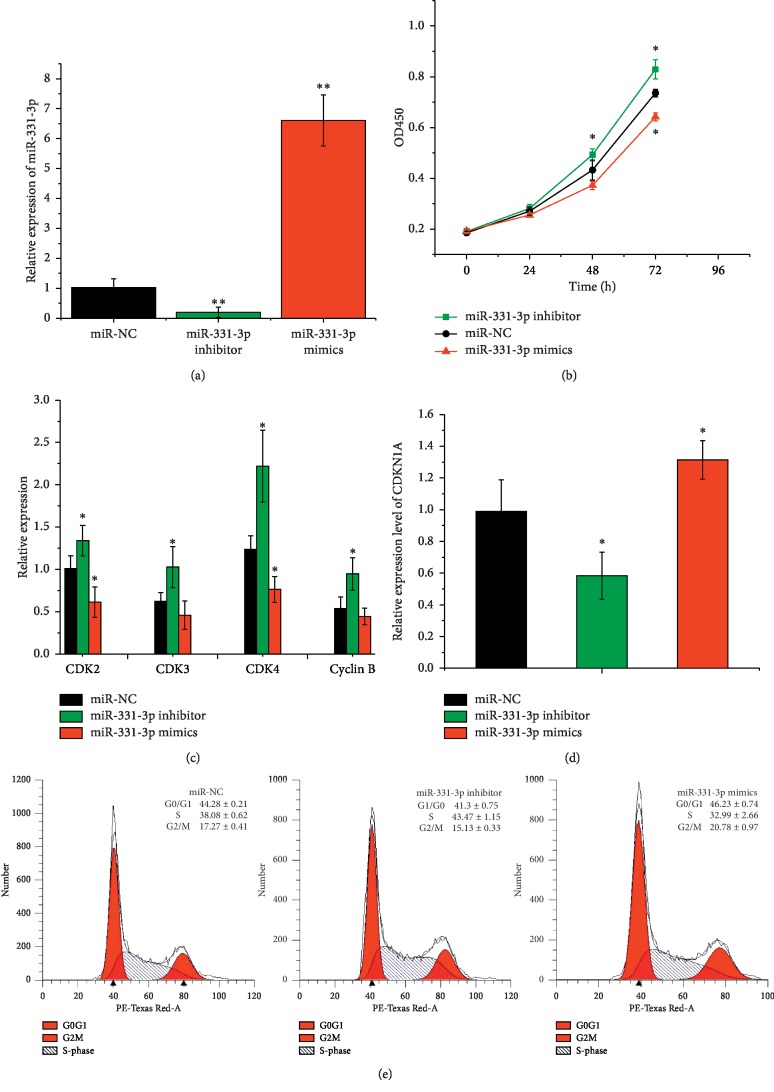
miR-331-3p suppressed the proliferation of porcine intramuscular preadipocytes. (a) Expression level of miR-331-3p measured by qRT-PCR. (b) Cell Counting Kit 8 (CCK-8) assays results of cell proliferation. (c) qPCR results of the relative expression levels of CDK2, Cyclin B, CDK3, CDK4, and (d) CDKN1A. (e) Results of cell cycle analysis by flow cytometry. Data represent means ± SD (^*∗*^*p* < 0.05; ^*∗∗*^*p* < 0.01).

Preadipocyte proliferation, differentiation, and fatty acid accumulation form the basis of adipogenesis. To elucidate whether miR-331-3p regulates adipogenesis, the effects of miR-331-3p on preadipocyte proliferation were first explored. Synthetic miR-331-3p mimics, miR-331-3p inhibitors, and the negative control (miR-NC) are shown in Figure 2(a), which were transfected into preadipocytes, respectively. Additionally, miR-331-3p mimics significantly increased the expression levels of miR-331-3p in preadipocytes while endogenic expression of miR-331-3p in preadipocytes was remarkably inhibited by transfecting with miR-331-3p inhibitors. Subsequently, cellular proliferation was detected by the addition of the dye, CCK-8. As presented in Figure 2(b), miR-331-3p expression significantly suppressed cell proliferation at 24 h, 48 h, and 72 h (*p* < 0.05). In contrast, the miR-331-3p inhibitor significantly promoted cell proliferation at 48 h and 72 h. To verify the accuracy of this result, a series of marker genes' expression were quantified (Figure 2(c)), including CDK2, CDK3, CDK4, and Cyclin B. These genes were significantly downregulated in the group receiving the miR-331-3p mimic. The mRNA expression of CDKN1A was significantly upregulated after transfection of miR-331-3p mimics (Figure 2(d)).

Moreover, the cell cycle distribution in miR-331-3p transfected preadipocytes was investigated. In comparison with miR-NC group, the miR-331-3p mimic treated group prominently increased the number of cells in G0/G1 phase; both of S phase and G2/M phase decreased significantly (*p* < 0.05, Figure 2(e)). Taken together, these data indicate that transfection with miR-331-3p could effectively increase the proliferation of adipocytes.

### 3.3. miR-331-3p Promoted Differentiation of Preadipocytes

**Figure 3:**
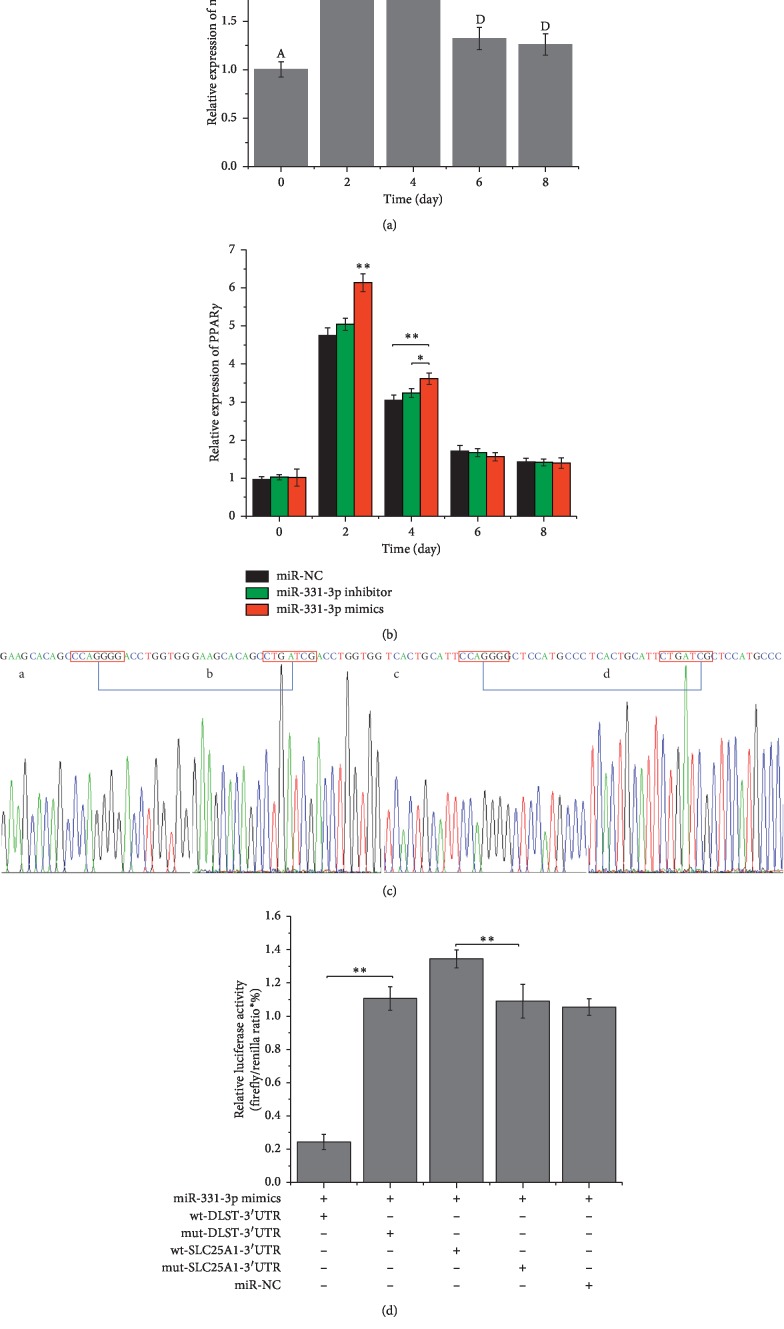
miR-331-3p expression during preadipocyte differentiation and confirmation of its target genes. (a) Level of miR-331-3p in preadipocytes differentiation process. (b) miR-331-3p influenced PPAR*γ* expression in preadipocytes differentiation process. (c) The seed region of DLST and SLC25A1 CCAGGG mutated to CTGATCG using overlapping PCR. (d) Two pmirGLO vector constructs, containing either the DLST-3′-UTR (or SLC25A1-3′-UTR) or the DLST-3′-UTR (or SLC25A1-3′-UTR) with a mutation in the miR-331-3p seed region, were transfected into PK-15 cells either alone or in combination with NC or miR-331-3p mimic. Renilla luciferase activity was normalized to firefly luciferase. Data represent means ± SD (^*∗*^*p* < 0.05; ^*∗∗*^*p* < 0.01). Different lowercase letters denoted significant difference (*p* < 0.05) and different capital letters represent very significant differences (*p* < 0.01).

In our previous research, we successfully constructed the eukaryotic expression vector of miR-331-3p (pre-miR-331-3p) [34]. To investigate the relationship between the expression of miR-331-3p and preadipocyte differentiation, preadipocytes induced differentiation. This day is defined as the 0th day. Expression levels of miR-331-3p were measured by qRT-PCR on days 0, 2, 4, 6, and 8. The level of miR-331-3p was investigated during porcine preadipocyte differentiation. miRNA expression profiling revealed that the level of miR-331-3p was upregulated firstly and then downregulated during adipogenesis of porcine preadipocyte cells (0 d–8 d) (Figure 3(a)). Even on day 8 after cell differentiation, the expression of miR-331-3p was still higher than that on day 0. In addition, preadipocytes were transiently transfected with pre-miR-331-3p, pcDNA 3.1(+), or miR-331-3p inhibitor prior to differentiation. Two days later, complete medium was replaced with differentiation induction medium. Expression of a marker gene for differentiation, PPAR*γ*, was detected (Figure 3(b)). The expression level of PPAR*γ* was upregulated in the pre-miR-331-3p group relative to the pcDNA 3.1(+) and miR-331-3p inhibitor treated groups. Therefore, it could be reasonably hypothesized that miR-331-3p may promote preadipocyte differentiation.

### 3.4. The Determination of Target Gene of miR-331-3p

To confirm target gene for miR-331-3p, 2 different bioinformatics software programs were used to predict the target genes. Interestingly, the 2 programs (Targetscan, miRWalk) produced different results. The genes were analyzed using gene ontology and pathway analysis to confirm the likely targets of miR-331-3p. It was determined that DLST and SLC25A1 were both associated with fatty acid metabolism and are components of the citric acid pyruvate cycle. To verify direct interactions between miR-331-3p and the 3′-UTR of DLST and SLC25A1, the 3′-UTR regions were cloned into a luciferase reporter vector (Figure 3(c)) and were used to transiently transfect PK-15 cells. Luciferase activity was observed after 2 days (Figure 3(d)). miR-331-3p mimics were cotransfected with wild-type DLST 3′-UTR vector, and luciferase activity was decreased relative to the NC or mut-type DLST 3′-UTR vector groups. However, the groups were cotransfected with miR-331-3p mimics and wild-type SLC25A1 3′-UTR vectors, and luciferase activity was increased relative to the NC or mut-type SLC25A1 3′-UTR vector groups. It can be concluded that DLST was the direct target gene of miR-331-3p, which may regulate DLST. Meanwhile, SLC25A1 is more likely to be an indirect target of miR-331-3p.

### 3.5. miR-331-3p Regulates the Gene Expressions of DLST and SLC25A1 in Porcine Preadipocytes

**Figure 4:**
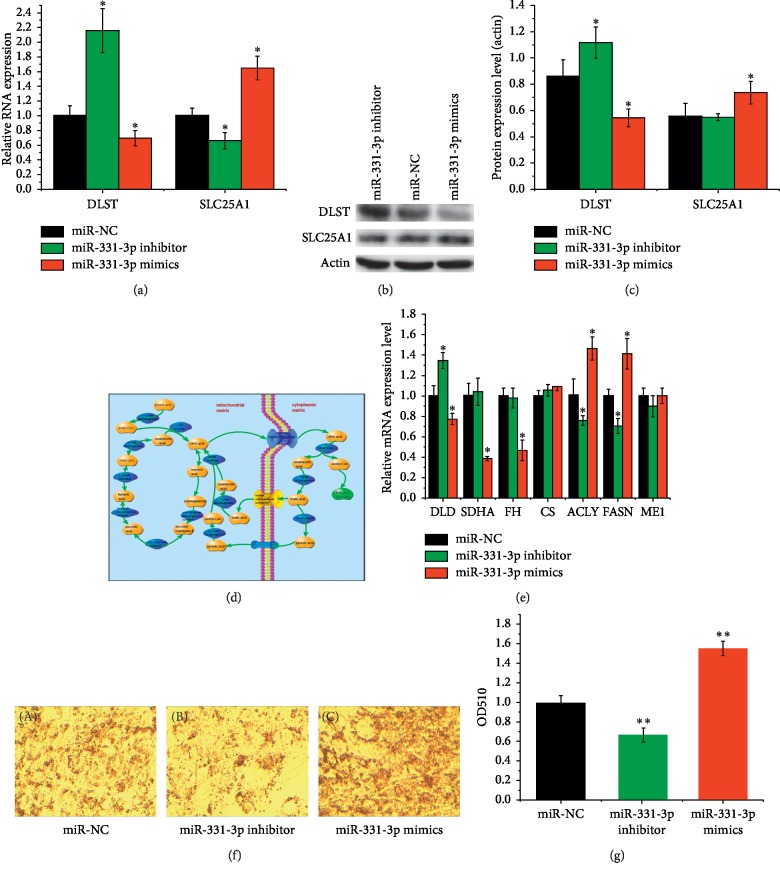
miR-331-3p can also promote the synthesis of fatty acids by regulating the target gene DLST to promote acetyl-CoA translocation. (a) The expression of DLST and SLC25A1 measured by qRT-PCR. (b) DLST and SLC25A1 protein were detected by Western blotting. (c) DLST and SLC25A1 protein expression were quantified. (d) DLST coding dihydrolipoamide succinyltransferase and SLC25A1 coding mitochondrial citrate transporter played a critical role in citrate pyruvate cycle. (e) Some genes coding enzymes of citrate pyruvate cycle were measured by qRT-PCR. (f) Images of cells stained with Oil Red O after miR-331-3p mimics, miR-331-3p inhibitor, and miR-NC transfection; (g) Quantification of fatty acid of adipocyte. Data represent means ± SD (^*∗*^*p* < 0.05; ^*∗∗*^*p* < 0.01).

To further study the mechanism by which miR-331-3p regulates DLST and SLC25A1 gene expressions, the miR-331-3p and RNA expression level of DLST and SLC25A1 were measured 2 days after transfection with miR-331-3p mimics, miR-NC, and miR-331-3p inhibitor. The gene expression of miR-331-3p was significantly upregulated (*p* < 0.05) in the experimental group and the contrary result was observed in the inhibitor group. Meanwhile, the expression of DLST was significantly downregulated (*p* < 0.05) in the experimental group and the contrary result was observed in the inhibitor group, but the expression of SLC25A1 had opposite tendency with the expression of DLST, which means that the gene expression of SLC25A1 was significantly upregulated in the mimics treatment group (*p* < 0.05) (Figure 4(a)).

To further clarify whether the miR-331-3p regulates the protein expression of DLST and SLC25A1, total protein was extracted for Western blot analysis. The level of DLST protein was downregulated in the miR-331-3p mimic group compared with the negative control and inhibitor groups (*p* < 0.05) (Figures 4(b) and 4(c)). In contrast, the protein expression of SLC25A1 in the mimics group was significantly upregulated compared with the inhibitor group (*p* < 0.05) and was also upregulated compared with the negative control (*p* < 0.05).

### 3.6. The Role of miR-331-3p in Adipocyte Fatty Acid Metabolism

Both DLST and SLC25A1 are important components of citrate pyruvate cycle pathway (Figure 4(d)). The DLST gene encodes the dihydrolipoamide succinyl transferase, a core enzyme of the mitochondrial *α*-ketoglutarate dehydrogenase complex (KGDHC). The KGDHC catalyzes *α*-ketoglutarate to form succinyl coenzyme A. The citrate pyruvate cycle not only results in the synthesis acetyl-CoA but also functions to transport it from the mitochondrial to the cell matrix. This is the only source of acetyl-CoA, which is indispensable for all fatty acid synthesis. To further study the mechanism of action, mRNA expressions of some known genes involved in the citrate pyruvate cycle were analyzed, the results of which are presented in Figure 4(e). The expressions of citrate lyase (CS), SLC25A1, ATP citrate lyase (ACLY), and fatty acid synthase (FASN) were upregulated in the miR-331-3p mimic treated group relative to the NC and inhibitor treated groups (*p* < 0.05). However, the expressions of DLST, dihydrolipoamide dehydrogenase (DLD), succinate dehydrogenase complex flavoprotein subunit A (SDHA), fumarate hydratase (FH), malic enzyme 1 (ME1), pyruvate carboxylase (PC), and malate dehydrogenase 1 (MDH1) were all downregulated in the miR-331-3p mimic treated group (*p* < 0.05).

Preadipocytes were transfected with either miR-331-3p mimic, miR-331-3p inhibitor, or miR-331-3p NC. The preadipocytes were then stained with Oil Red O (Figure 4(f)), and fatty acid contents of the cells were assessed (Figure 4(g)). It can be clearly seen that the fatty acid content of the miR-331-3p mimic treated group is substantially higher than that in the other treatment groups (*p* < 0.05).

## 4. Discussion

In the present study, miR-331-3p was identified as a novel gene that regulates proliferation, differentiation, and fatty acid accumulation of porcine preadipocyte. The organization of miR-331-3p in Laiwu and Yorkshire pigs was analyzed. The expression of this miR-331-3p was observed to be consistent with the previously published sequencing results [30, 31], which is suggestive of the important role it plays in the regulation of fat formation. The expression of miR-331-3p in different tissues was also analyzed. Compared with Yorkshire pigs, the expression of miR-331- 3p was significantly elevated in the liver, the muscle, and the back fat of Laiwu pigs. The liver is an important organ for lipid metabolism and plays an important role in the digestion, absorption, synthesis, decomposition, and transport of lipids [35, 36]. Fat is the main tissue of lipid storage. The free fatty acids produced by the hydrolysis of triglycerides in fat can be transported to the tissues through oxidative decomposition and energy supply after being combined with plasma albumin [37]. The muscle itself contains adipose tissue, which is referred to as intramuscular fat. The intramuscular fat mainly refers to the fat deposited among the fibers in the muscle connective tissue and the muscle bundle. Intramuscular fat has certain specificities in lipid metabolism and cell differentiation, which is related to the special location of IMF [38]. It has been well established that Yorkshire pigs are the leanest breeds, and its liver, back fat, and muscle are important sites for the proliferation and differentiation of preadipocytes and significant on fatty acid accumulation. Therefore, the high expression of miR-331-3p observed in Laiwu pigs may be due to the fact that miR-331-3p has the ability to orchestrate these processes.

Two candidate target genes of miR-331-3p were predicted through a biological website analysis, and its expression levels were assessed in various tissues between the 2 breeds of pigs by qRT-PCR. Compared with Yorkshire pigs, the expression of DLST was significantly reduced in the liver, the muscle, and the back fat of Laiwu pigs. However, except in liver tissue, the expression of SLC25A1 was significantly elevated in muscle and back fat. This may indicate that there is a negative regulatory targeting relationship between miR-331-3p and DLST, but a positive regulatory targeting relationship between miR-331-3p and SLC25A1 was uncertain.

The formation of adipose tissue is a process in which preadipocytes grow, proliferate, undergo terminal differentiation to form fat cells, and grow by hypertrophy. In the present study, preadipocytes were isolated from Laiwu pigs. Above all, CCK8 assay and flow cytometry analysis were used to detect cell growth when preadipocytes were transfected with miR-331-3p mimics, miR-331-3p inhibitors, or miR-NC. Results of CCK8 assay showed that cell proliferation rate of cells when transfected with miR-331-3p mimics was significantly lower than that in cells transfected with miR-331-3p inhibitors, or miR-NC, and flow cytometry analysis showed that after transfection with miR-331-3p mimics, the proportion of cells in G0/G1 phase increased significantly, while the proportion of cells in S phase and G2/M phase decreased significantly compared with cells transfected with miR-331-3p inhibitor and miR-NC. These results were consistent with the results of CCK8 analysis, which indicated that miR-331-3p could inhibit cell proliferation. In order to further elucidate the mechanism of action, qRT-PCR was used to detect several proliferation-related genes, including CDK2, CDK3, CDK4, Cyclin B, and CDKNIA. Cyclin-dependent kinases (CDKs), such as CDK2, CDK3, and CDK4, have been recognized as key regulators of cell growth and proliferation in eukaryotic cells and are required for G1-S phase transitions in mammalian cells [39, 40]. Cyclin B is required for cells to enter and exit the M phase of the cell cycle [41]. Conversely, overexpression of cellular CDKN1A may be an inhibitor of cell proliferation [42]. Levels of CDK2, CDK3, CDK4, and Cyclin B in preadipocytes transfected with miR-331-3p were significantly lower than those in cells transfected with miR-NC; however, the difference of CDKN1A expression is contrary to that of other genes. Therefore, miR-331-3p may inhibit the cells by affecting the expression of cell cycle-related genes. Taken together, these data indicate that miR-331-3p is likely an inhibitory regulator of the proliferation of porcine adipocyte progenitor cells.

Subsequently, overexpression of miR-331-3p was able to induce differentiation of preadipocytes. Furthermore, miR-331-3p expression was detected at days 0, 2, 4, 6, and 8 of cell differentiation. Trends in expression were increasing initially and then gradually decreasing. The expression of the PPAR*γ* gene also showed the same trend. The PPAR family of transcription factors is important regulators of the differentiation of precursor adipocytes into mature adipocytes. The PPARs family contains 3 subtypes, alpha, beta, and gamma. Among them, PPAR*γ* is the most important marker gene in the process of precursor adipocyte differentiation [43]. The expression of the PPAR*γ* gene was increased significantly on days 2 and 4 after the overexpression of miR-331-3p. The expression trend of binding to miR-331-3p is suggestive of a role in the promotion of the differentiation process in preadipocytes.

Genes related to fatty acid metabolism (DLST and SLC25A1) were predicted bioinformatically. Next, these predicted target genes were verified experimentally by a Dual-Luciferase Reporter Assay. Luciferase activity of wt-DLST-3′-UTR was significantly suppressed by ectopic expression of miR-331-3p. However, luciferase activity of wt-SLC25A1-3′-UTR was significantly upregulated. This may be due to the fact that the SLC25A1 gene is not a direct target of miR-331-3p; rather it may regulate the expression indirectly by other means. The results of qRT-PCR and Western blot of DLST and SL25A1 genes also proved this conclusion. After transfection of miR-331-3p mimics, miR-NC, and miR-331-3p inhibitor, induced mature adipocytes were stained by Oil Red O and its lipid droplet quantified. Those results showed that overexpression of miR-331-3p indeed increased fatty acid synthesis, which in turn corroborates the hypothesis that miR-331-3p can promote fatty acid synthesis through the modulation of the citrate pyruvate cycle.

## 5. Conclusions

In summary, miR-331-3p functions as a regulator of the proliferation and differentiation of adipocytes and fatty acid metabolism. Overexpression of miR-331-3p can inhibit cellular proliferation and was observed to promote preadipocytes differentiation. Furthermore, miR-331-3p can also promote the synthesis of fatty acids by regulating the target gene DLST to promote acetyl-CoA translocation.

## Figures and Tables

**Table 1:** Primer sequences used for the construction of luciferase reporter plasmids: Oil Red O staining and quantification of fatty acid.

Target	Primer sequence (5′–3′)
F1	AAGGTACCGCCTTTCCTTCATTAG
R1	AACTCGAGAACAATGTGGTAGGAT
F2	AAGGTACCTAAGATACTACGATGT
R2	AACTCGAGTTTATTCTGCCTTGGA
F3	AAGCAC AGCCTGATCG ACCTGGT
R3	ACCAGGTCGATCAGGCT GTGCTT
F4	TCACTGCATTCTGATCGCTCCATGC
R4	GCATGGAGC GATCAGAATG CAGTGA

**Table 2:** Primer sequences used for quantitative real-time polymerase chain reaction and gene cloning.

Gene	GenBank accession no.	Primer sequence (5′–3′)
DLST	NC_010449.5	AGCCCCAAAAGCAGAACC
		GGGCAGCAGTGGGTTTTA
SLC25A1	NC_010456.5	TGACCAGACCTCTTCCAA
		AACGAAGAAGCGGATGGC
GAPDH	NC_010447.5	AGGTCGGAGTGAACGGATTTG
		ACCATGTAGTGGAGGTCAATGAAG
CDK2	NC_010447.5	AAGATGGACGGAGCTTGTTATCGC
		CTGGCTTGGTCACATCCTGGAAG
CDK3	NC_010454.4	TCATCCACCGAGACCTGAAGCC
		AGACATCCACAGCCGTCGAGTAG
CDK4	NC_010447.5	TGAGATGGAGGAGTCTGGAGCAC
		CTCGGAAGGCAGAGATTCGCTTG
Cyclin B	NC_010458.4	GACTGGCTAGTGCAGGTTCAGATG
		ATGGCAGTGACACCAACCAGTTG
CDKN1A	NC_010449.5	CGAGAGCGATGGAACTTCGACTTC
		TCCACATGGTCCTCCTGAGACG
PPAR*γ*	NC_010455.5	AGGACTACCAAAGTGCCATCAAA
		GAGGCTTTATCCCCACAGACAC
CS	NC_010447.5	ACACTCAACTCAGGACGGGT
		TTCCAGGAGGACATTGGGCA
ACLY	NC_010454.4	CTTGATCCGCAAACCTGCCT
		CCGTCACCATCAGGCACATC
DLD	NC_010451.4	GGCGCAGTGCACATTGACTA
		ACCATGCCATCTGTGTCAGC
SDHA	NC_010458.4	CCTGGAGTTCGTGCAGTTCC
		AACCTCTCACCCTGGCTGTT
FH	NC_010452.4	TGGTGCCCAGACTGTGAGAT
		AGCTGCACGCTTCAAGATCC
ME1	NC_010443.5	TTGCAGCCCTTCGAATCACC
		AGGTGTGCAATCCCTAAGGC
PC	NC_010444.4	AGAACGAGATCCCAGGAGGC
		GACTCAGCCCATTCTGCACC
MDH1	NC_010445.4	CATGCCAAGAAGGGATGGCA
		CCTTGGGAATGGATGGAGCC

## Data Availability

The data used to support the findings of this study are available from the corresponding author upon request.
